# The Relationship Between Microbial Community Structures and Environmental Parameters Revealed by Metagenomic Analysis of Hot Spring Water in the Kirishima Area, Japan

**DOI:** 10.3389/fbioe.2018.00202

**Published:** 2018-12-20

**Authors:** Eri Nishiyama, Koichi Higashi, Hiroshi Mori, Konomi Suda, Hitomi Nakamura, Soichi Omori, Shigenori Maruyama, Yuichi Hongoh, Ken Kurokawa

**Affiliations:** ^1^Biotechnological Research Support Division, FASMAC Co. Ltd, Kanagawa, Japan; ^2^Department of Biological Information, Tokyo Institute of Technology, Tokyo, Japan; ^3^Center for Information Biology, National Institute of Genetics, Shizuoka, Japan; ^4^Institute for Geo-Resources and Environment, National Institute of Advanced Industrial Science and Technology, Ibaraki, Japan; ^5^Department of Solid Earth Geochemistry, Japan Agency for Marine-Earth Science and Technology, Yokosuka, Japan; ^6^Faculty of Liberal Arts, The Open University of Japan, Chiba, Japan; ^7^Department of Earth and Planetary Sciences, Tokyo Institute of Technology, Tokyo, Japan; ^8^School of Life Science and Technology, Tokyo Institute of Technology, Tokyo, Japan

**Keywords:** extreme environments, environmental parameters, microorganisms, metagenome, hot springs

## Abstract

Diverse microorganisms specifically inhabit extreme environments, such as hot springs and deep-sea hydrothermal vents. To test the hypothesis that the microbial community structure is predictable based on environmental factors characteristic of such extreme environments, we conducted correlation analyses of microbial taxa/functions and environmental factors using metagenomic and 61 types of physicochemical data of water samples from nine hot springs in the Kirishima area (Kyusyu, Japan), where hot springs with diverse chemical properties are distributed in a relatively narrow area. Our metagenomic analysis revealed that the samples can be classified into two major types dominated by either phylum Crenarchaeota or phylum Aquificae. The correlation analysis showed that Crenarchaeota dominated in nutrient-rich environments with high concentrations of ions and total carbons, whereas Aquificae dominated in nutrient-poor environments with low ion concentrations. These environmental factors were also important explanatory variables in the generalized linear models constructed to predict the abundances of Crenarchaeota or Aquificae. Functional enrichment analysis of genes also revealed that the separation of the two major types is primarily attributable to genes involved in autotrophic carbon fixation, sulfate metabolism and nitrate reduction. Our results suggested that Aquificae and Crenarchaeota play a vital role in the Kirishima hot spring water ecosystem through their metabolic pathways adapted to each environment. Our findings provide a basis to predict microbial community structures in hot springs from environmental parameters, and also provide clues for the exploration of biological resources in extreme environments.

## Introduction

The increasingly widespread use of next-generation sequencing (NGS) has led to more information on the taxonomic composition of microorganisms from various environmental samples being obtained (Sun et al., 2014; Castelino et al., 2017). In particular, many hitherto unexamined microbial communities have been discovered in extreme environments, such as hot springs and deep-sea hydrothermal vents, and it has been reported that many of these microbes specifically live in extreme environments (Song et al., 2013; Tiago and Veríssimo, 2013; Chan et al., 2015; Zhang et al., 2016). It is assumed that microorganisms living in extreme environments have evolved various metabolic energy systems adapted to specific environmental factors derived from the local chemical milieu. Several studies of extreme environments, such as chimney 4143-1 in the Mothra hydrothermal vent field (Xie et al., 2011) and Yellowstone National Park (YNP) in the USA (Inskeep et al., 2010, 2013a; Jay et al., 2016) have reported specific microbial communities and their gene repertoires along with the environmental data. Recent studies have also revealed that specific environmental factors (e.g., temperature and sulfide concentrations) are related to microbial composition in the Philippines (Huang et al., 2013), the Tibetan Plateau in northwestern China (Wang et al., 2013), and in the Tengchong in southwest China (Hou et al., 2013). Research into microorganisms and environmental data from the YNP metagenome project reported that pH is one of important factors in forming the microbial community type in a hot spring environment (Inskeep et al., 2013b). There are limited studies discussing the kind of environmental factors that affect these community structures and their gene repertoires, as well as methods of predicting microbial taxon abundances from environmental factors.

Investigation of microorganisms living in extreme environments also plays a vital role in exploring biological resources. Various useful enzymes and bioactive compounds have been discovered from microbes derived from extreme environments, and have been applied to multiple fields, such as medicine and food production (Littlechild, 2015; Mahajan and Balachandran, 2017). If we can predict what kind of microbes live in an extreme environment from the environmental factors, this will lead to a more efficient search for useful biological resources. To establish a method for predicting the microbial community structure from environmental factors, it is essential to compare the community structures among multiple sampling points showing the diverse properties of environmental factors. An environment suitable for such comparative analysis is in the Kirishima area of Japan.

**Figure 1:**
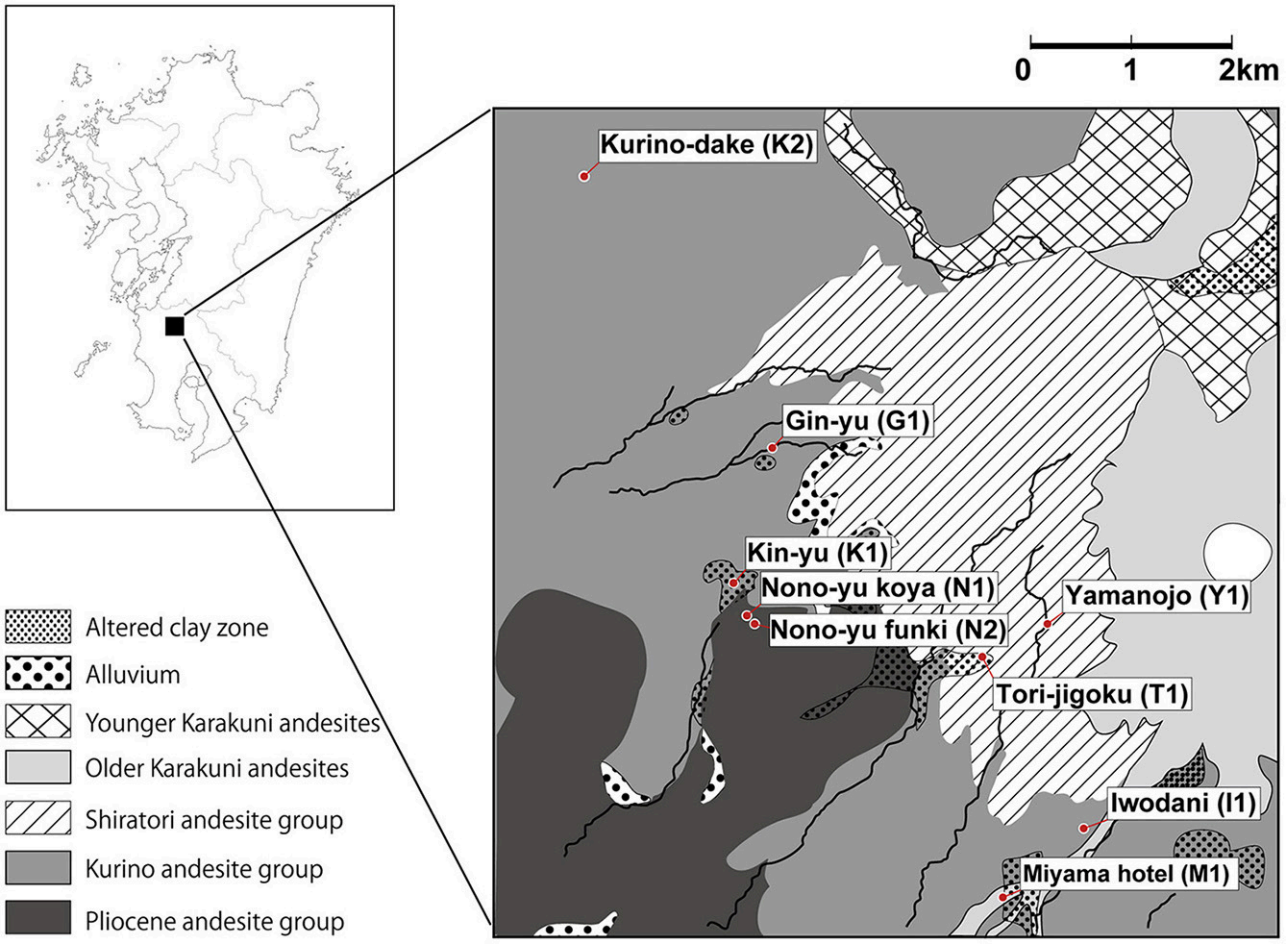
Geological map of the Kirishima area showing locations of the sampling sites for metagenomic analysis.

Many hot springs occur in the area where the volcanic front crosses the Kyushu region of Japan. The Kirishima area is located in the Kyushu region and has hot springs with various chemical properties distributed in a relatively narrow area (Figure 1 and Supplementary Figure 1; Supplementary Table 1). The Kirishima area is mainly composed of andesite, and hot spring water from two origins is found there (Gokou et al., 1995): (1) geothermal fluid gushing from the surface of the earth through a fault at a depth of 2 km; and (2) high-temperature fluid originating from surface water and circulating at a depth of about 1 km. The hot spring water in the Kirishima area has various chemical properties, depending on the mixing ratio of the two types of water. For example, water with a low mixing ratio of volcanic gas and produced by heating and circulating surface water mainly at depth, has a pH close to neutral. Hot spring water produced by mixing high-temperature volcanic gas and surface water near the surface of the earth becomes acidic.

Thus, the wide variety of hot springs in the Kirishima area—and their relatively easy accessibility—allows many environmental parameters to be measured and to provide useful information. Samples from the area are suitable for investigating the relationships between the microbial community composition and environmental factors of the hot springs. Although one example of the microbial communities in the Kirishima area has been reported, the study targeted only the archaeal communities in acidic regions (Satoh et al., 2013).

We focused on the Kirishima area as a general model of a microbial ecosystem in a hot spring environment and performed metagenomic analysis in this environment, along with measurement of environmental data (metadata) of each water sample from a hot spring. We also constructed regression models which predicted the abundance of dominant phyla living in the Kirishima hot springs, using these metadata. Based on the verification of the model parameters and the results of the gene enrichment analysis of metagenomic data, we examined the relationship between environmental parameters and the metabolic features of each water sample.

## Materials and Methods

### Sample Collection and DNA Preparation

The nine sampling sites were located in the Kirishima area in the central part of Kagoshima prefecture in Kyushu, Japan. All water samples from the hot springs were collected on two occasions: February 2012 and March 2015. To extract DNA for metagenomic sequencing, 1 L of each sample was filtered through a 0.2-μm pore size polycarbonate membrane. DNA was extracted from the materials on the filter membranes, using the Rapid Water DNA Isolation kit (QIAGEN) with bead homogenization on a Micro Smash MS-100R (TOMY) at 3,500 rpm for 20 s at 4°C.

### Physicochemical Analysis and Microbial Cell Counts

For the 2012 and 2015 samples, water temperature, pH, dissolved oxygen, electrical conductivity and salinity were measured onsite using a portable water-quality meter (P30 series, DKK-TOA Corporation). Water for chemical analysis was collected in 1 L polyethylene bottles and stored at 4°C. Both the metal and ion concentrations of water samples were measured after filtration (GF/F; pore size = 0.7 μm; Whatman). To determine the metals and major cations, 10 mL of water was acidified with HNO_3_ (pH < 2) and then analyzed by inductively coupled plasma-atomic emission spectroscopy (ICP-AES) (ICPS8100, Shimadzu). To determine the major anions, another 10 mL of water was subsampled and analyzed by ion chromatography (Dionex Qic analyzer) equipped with an IonPAC AS11-HC column (Dionex). Some ions and turbidity from the 2015 samples were measured using a water-quality meter (DPM-MT, KYORITSU CHEMICAL-CHECK). The concentrations of total carbon, total organic carbon (TOC), dissolved organic carbon and particulate organic carbon in the water samples were measured with a TOC-VCSH analyzer (Shimadzu). The concentrations of total nitrogen (TN), total organic nitrogen (TON), dissolved organic nitrogen and particulate organic nitrogen were measured with a U1800 spectrophotometer (Hitachi).

For microbial cell counts, 20 mL of water from a hot spring was filtered using a 0.2-μm pore size black polycarbonate membrane, and cells were stained with ethidium bromide and counted under a Nikon Eclipse 80i fluorescence microscope equipped with a Nikon Digital Sight DS-5Mc using the fluorescence filter GFP-L (excitation filter 510–560 nm, dichromatic mirror 575 nm, barrier filter 515 nm).

### Metagenomic Sequencing

To obtain sufficient DNA for metagenomic sequencing, the DNA samples prepared in 2012 were processed using the Nextera DNA Sample Prep kit (Illumina) for library construction with the following modifications of the manufacturer's instructions: addition of two polymerase chain reaction (PCR) cycles and alkaline denaturation after evaporation. Metagenomic sequencing of the libraries from the 2012 samples was performed using the Illumina GAIIx and MiSeq platforms (all in paired-end mode).

### Quality Filtering and Assembly of Illumina Sequence Reads

We discarded Illumina sequence reads that: (1) contained ambiguous nucleotides; (2) were mapped to the PhiX genome sequence by using Bowtie 2 version 2.1.0 with default parameters (Langmead and Salzberg, 2012); and (3) were identified as duplicates by comparing the first 30 nucleotides of reads. We then removed the adapter sequences in the reads using the cutadapt version 1.2.1 (Martin, 2011) and also removed low-quality 3′-end regions with a Phred-like quality score < 17. We also discarded reads with < 50 nucleotides, with an average Phred-like quality score < 25, or with no paired reads. After the quality filtering of sequence reads from all samples, we assembled the reads using the Platanus assembler version 1.2.1 (Kajitani et al., 2014).

### Identification and Taxonomic Assignment of 16S rRNA Gene Sequences

The 16S rRNA genes from metagenomic sequences were identified by examining metagenomic sequence reads by BLASTN searches against the SILVA non-redundant (nr) small-subunit rRNA gene sequence database (SILVA NR SSU rRNA DB) version 115 (Quast et al., 2013) with an E-value of < 1e^−8^, identity >85% and alignment length >50 bp. The resulting sequences were taxonomically assigned at the phylum level or lower taxonomic ranks, and the abundance of assigned reads was calculated.

### Gene Prediction, Functional Annotation and Coverage Calculation

Protein-coding sequences (CDSs) in the scaffolds were predicted using MetaGeneMark (Zhu et al., 2010). Functional annotation of the predicted CDSs was performed by a BLASTP (version 2.2.26) search against the Kyoto Encyclopedia of Genes and Genomes (KEGG) protein database (Kanehisa et al., 2014) (March 2014) with an *E*-value of < 0.01, identity >40% and score >70. The classification and abundance calculation of genes based on the KEGG Orthology was carried out by dividing the number of reads assigned to each KEGG ortholog (KO) by the median length of amino acid sequences in the corresponding KO in the KEGG protein database. To obtain more information on functions we performed a BLASTP search using all the predicted CDSs as queries against the GenBank nr database (May 2014) with an *E*-value of < 0.01.

### Analysis of Gene Enrichment in the Metagenome

From the KEGG protein database we chose prokaryotic genomes, one per genus, as a virtual reference microbiome (Supplementary Table 2). Enrichment scores were calculated by dividing the relative abundance of each KO in the Kirishima metagenomes by the average relative abundance of the corresponding KO in the virtual reference metagenome. To identify genes that were characteristically enriched in the Kirishima metagenomes, we selected genes with higher enrichment scores compared with average scores of the 35 universal single-copy genes (USCGs) and *gyrB* (Kato et al., 2015).

### Statistical Analysis

Principal component analysis (PCA) was performed using the function in the scikit-learn library (Pedregosa et al., 2011). For PCA using the relative abundance information of phylum- or genus-level taxonomic composition of the nine samples, the values of each feature (relative abundance of each taxon) were not scaled and used for the transformation as it was. For PCA using the KEGG module abundance data, KO abundance data and the measured values of 59 types of environmental parameters (two environmental parameters for which the measurement values were below detection limits in all samples were excluded), the values of each feature were first standardized as Z-scores and used for the transformation. To investigate the relationship between the coordinates of samples on the PCA plot and environmental parameters, the correlations between the principal component scores of samples and the measured values of each environmental parameter were calculated by the Pearson correlation coefficients using the SciPy library (Jones et al., 2001).

To detect the quantitative relationship between environmental parameters and microbial community structures, we constructed statistical models to predict the relative abundance of either Aquificae or Crenarchaeota from the measured values of environmental parameters. We performed principal component regression, which combines PCA with regression analysis, to avoid the multicollinearity problem caused by using a large number of explanatory variables. (1) The table of Z-score-converted environmental parameters was transformed to the table of principal component scores by performing PCA. By selecting only a subset of the obtained principal components, a small number of uncorrelated explanatory variables could be used for the regression model. Because the cumulative contribution rate of the first four principal components was more than 80%, these four principal components were used for regression analysis. (2) We constructed generalized linear models with a logit link and the binomial family, using all combinations (15 patterns) of these four principal components as explanatory variables, and compared the performance of these models by using Akaike's Information Criterion (AIC) (Aho et al., 2014) (Supplementary Table 3). The estimation of generalized linear models was done using the Statsmodels Python modules (Seabold and Perktold, 2010). (3) The models with minimum AIC values were selected, and we examined the environmental parameters affecting the relative abundances of phyla by inspecting the factor loadings obtained by the initial PCA.

## Results and Discussion

### Physicochemical and Biological Properties of the Kirishima Hot Spring Samples

Supplementary Table 4 presents the physicochemical and biological characteristics of the nine water samples from hot springs in the Kirishima area in 2012 and 2015 (Supplementary Table 1). We measured the water-quality data of 5 physical properties (e.g., total bacterial counts, turbidity, pH), 11 chemical properties (e.g., total carbon, TN), 10 anion species and 35 cation species, totaling 61 types (Supplementary Table 4).

Samples of G1, T1, K2, N2, and I1 from a hot spring with high water turbidity showed low pH; high concentrations of sulfate, Cu and Zn ions; and relatively high TOC and TN, in comparison to transparent and medium pH samples, such as K1, Y1, N1, and M1 (Supplementary Table 4 and Supplementary Figure 1). The total cell counts were as low as 1.21 × 10^4^-4.03 × 10^6^ cells mL^−1^, as reported in YNP (Meyer-Dombard et al., 2005). In summary, the temperatures of the samples were similarly high (84.0–96.8°C) except sample N1 (68°C), but they showed diverse chemical properties within this narrow area; thus these samples were suitable for use in analyzing the relationship between microbial community structures and environmental parameters.

### Metagenomic Sequencing and Taxonomic Composition of the Kirishima Hot Spring Microbiomes

Because it was difficult to extract sufficient DNA from the Kirishima hot spring water samples with low cell density, we used a PCR-based method to prepare sequence libraries for the nine hot spring samples. Metagenomic sequences were obtained using the Illumina platforms, and standard quality filtering yielded 1,965,768–54,065,035 read pairs for each of the nine samples. We then assembled the reads to construct contigs and scaffolds (Supplementary Table 5). The total number of scaffolds >500 bp in length was 4,019–92,715, and the largest scaffold size was 493,449 bp in the K1 sample (Supplementary Table 5).

**Figure 2:**
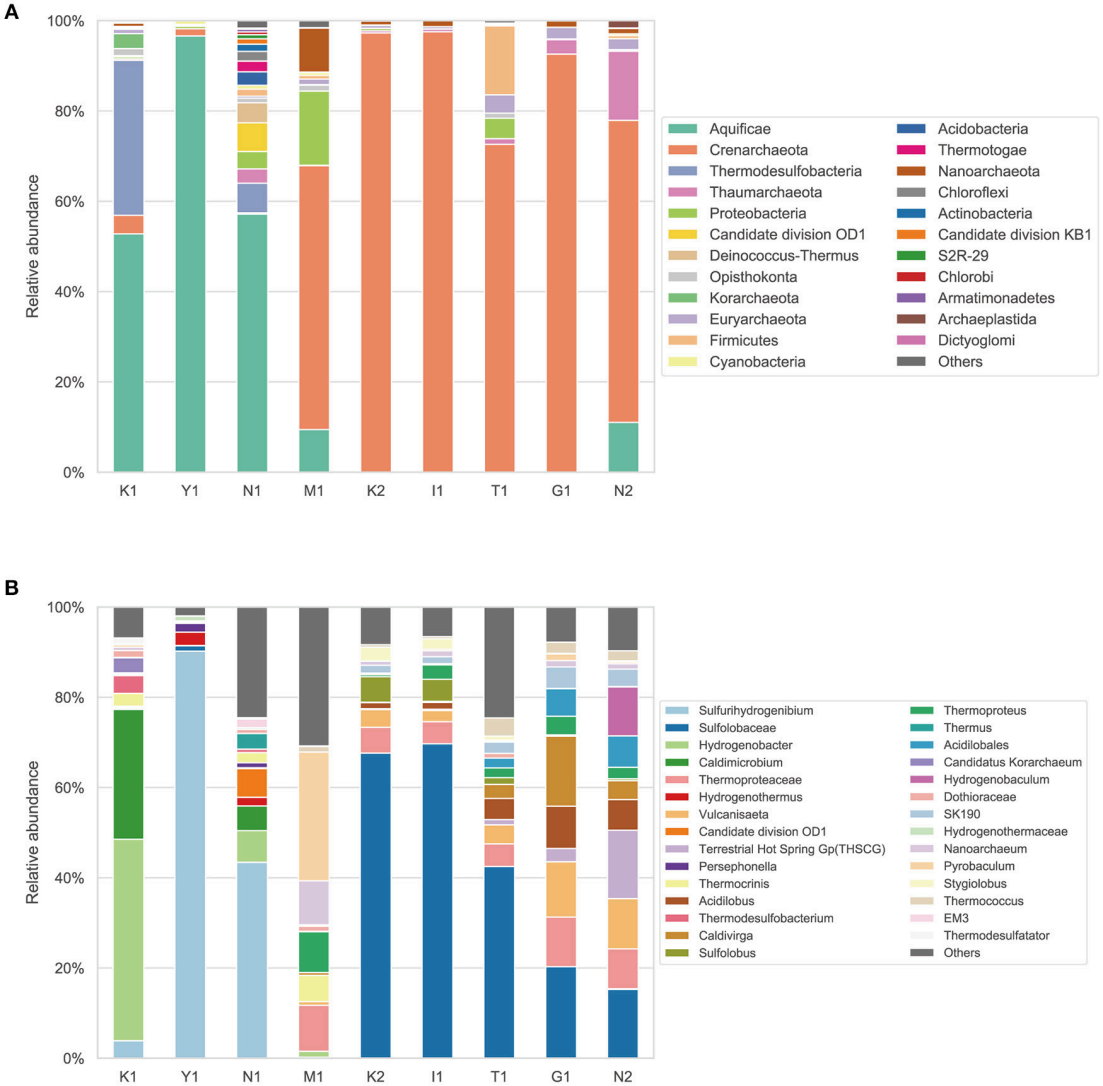
Taxonomic composition and relative abundance of microbiota in water from Kirishima hot springs based on 16S rRNA genes identified from the metagenomic reads. **(A)** Taxonomic composition of the microbiota at phylum level. **(B)** Detailed taxonomic composition (i.e., genus, family or order) of the microbiota.

The phylum- and lower-level taxonomic composition for each sample was examined by identifying 16S rRNA genes from the standard quality filtered metagenomic reads (Figure 2). In the nine hot spring samples, 10 prokaryotic phyla accounted for >80% of total taxonomically assigned reads, and either Aquificae or Crenarchaeota predominated. The Aquificae accounted for 52.7–96.6% of the taxonomically assigned reads in samples K1, Y1, and N1. The Crenarchaeota accounted for 52.5–96.6% in samples M1, K2, I1, T1, G1, and N2 (Figure 2A). The genus *Sulfurihydrogenibium* of the Aquificae dominated in Y1 and N1, and the family Sulfolobaceae of the Crenarchaeota dominated in K2, I1, T1, G1, and N2 (Figure 2B). This result was consistent with the previous report that archaea belonging to the order Sulfolobales were detected in samples (Pond-A and Pond-B) with higher dissolved element concentration in the Kirishima hot springs (Satoh et al., 2013). One isolate of the Sulfolobaceae from an acidic hot spring, *Sulfodiicoccus acidiphilus*, showed adaptation to low pH and high temperature (Sakai and Kurosawa, 2017).

The phylum Thermodesulfobacteria was also dominant in sample K1 (34.2% of the taxonomically assigned reads) and accounted for 6.5% in N1. The phyla candidate division OD1 (“Parcubacteria”) and “Deinococcus-Thermus” were the third and fourth most dominant in sample N1, although other diverse phyla were also detected. The phylum Cyanobacteria was detected in seven of the nine samples, albeit with < 1% relative abundance. The phylum Euryarchaeota was found in all samples with a low abundance (0.02–4.1%). These results showed confined phylogenetic diversity of the microbiomes in the water from the Kirishima hot springs with the predominance of either Crenarchaeota or Aquificae.

**Figure 3:**
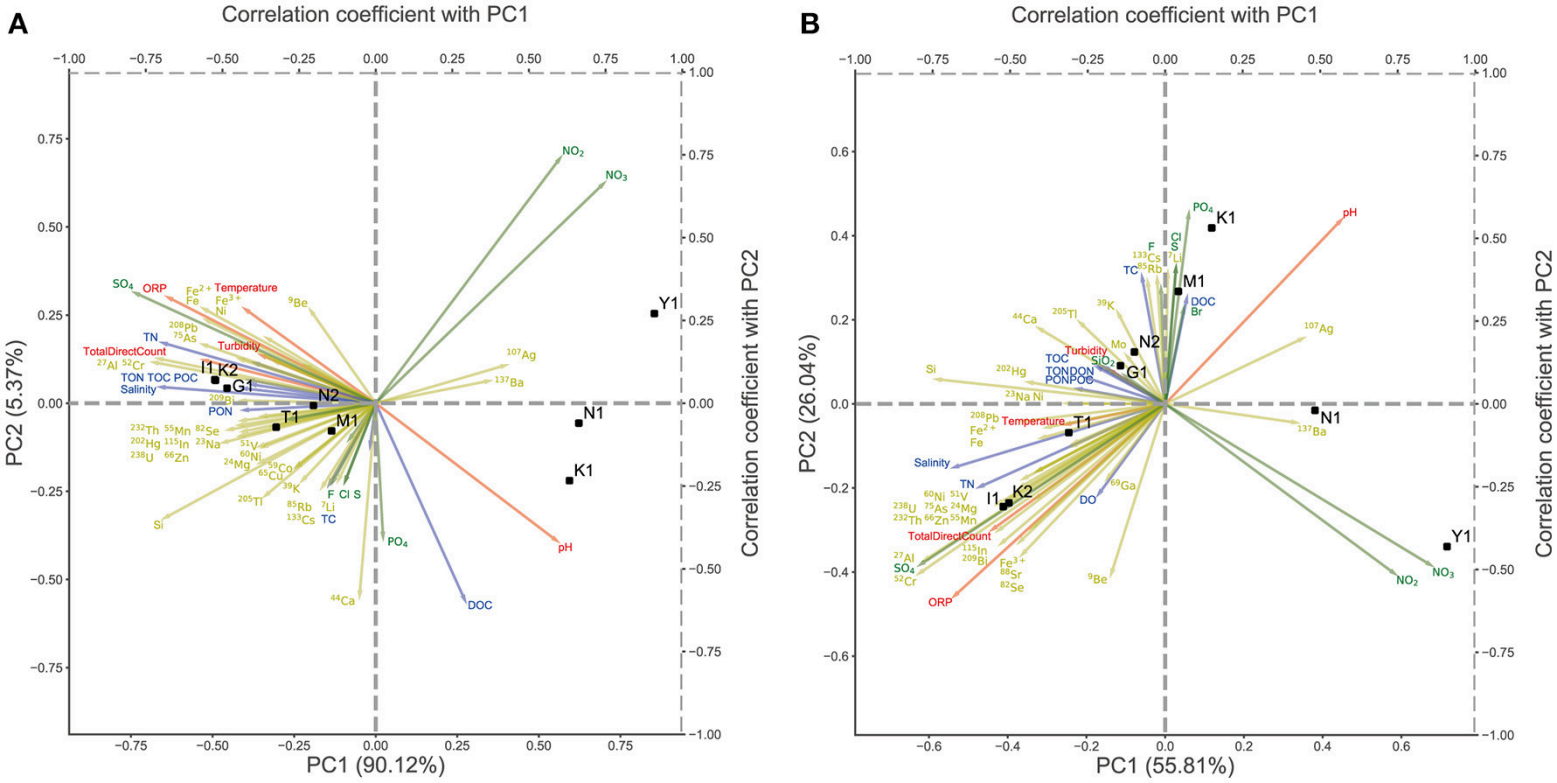
Principal component analysis (PCA) of the relative abundance of each sample across nine sites. The arrows represent the correlation coefficient between the principal component scores and each environmental parameter. The yellow arrows represent cations, the green arrows represent anions, the blue arrows represent water quality and the red arrows represent physical properties. **(A)** PCA of the relative abundance at phylum level. **(B)** PCA of the relative abundance at detailed taxonomic level.

To compare the taxonomic compositions among these microbiomes, we performed PCA using the relative abundance data of the phylum- and lower-level taxonomic composition (Figure 3). PCA showed that the first two principal components (PC1 and PC2) accounted for more than 95% of the variation in taxonomic composition among samples, and samples clustered into two major groups [(K1, Y1, and N1) vs. (M1, K2, I1, T1, G1, and N2)], each dominated by either Aquificae or Crenarchaeota, respectively. PCA of the lower taxonomic composition showed similar results, except for the position of sample K1, which was closer to M1 than to N1 and Y1 (Figure 3B). Next, to identify the environmental factors affecting the formation of these two major groups, we calculated the correlations between the principal component scores of all samples and the measured environmental data of each. In Figure 3, the positions indicated by the arrows represent the correlation coefficients between the measured values of environmental factors and the PC1 and PC2 scores. Most cations and TN show negative correlations with the PC1 scores and are densely plotted on the left-hand side of the plot, where samples dominated by Crenarchaeota (M1, K2, I1, T1, G1, and N2) are located. However, few environmental factors were positively correlated with the PC1 scores, so there are few arrows on the right-hand side of the plot where samples dominated by Aquificae (K1, Y1, and N1) are located. Sulfuric acid (SO_4_ in Figures 3A,B) showed a strong negative correlation with PC1 and appeared to contribute to the variation in community structures, consistent with a previous report (Huang et al., 2013). These results suggested that the differences in community structures reflect the differences in environmental parameters among samples.

### Construction of a Model for Estimating Microbial Abundance From Environmental Parameters

We constructed multiple regression models to predict the relative abundance of the two dominant taxa, either Aquificae or Crenarchaeota, using 59 types of environmental data as explanatory variables. When two or more explanatory variables are highly correlated, the regression coefficient estimate becomes unstable (this is known as the multicollinearity problem). To overcome it, we performed principal component regression (see Statistical Analysis in Materials and Methods). We selected a model using PC1 and PC3 as explanatory variables for the prediction model of Aquificae abundance, based on the minimum AIC value (the “best” model) (Supplementary Table 3 and Supplementary Figure 2A). For the prediction model of Crenarchaeota abundance, a model using PC1, PC3, and PC4 as explanatory variables exhibited the minimum AIC value (Supplementary Table 3 and Supplementary Figure 2B). In the prediction model for Aquificae, the regression coefficients were positive for PC1 and negative for PC3, which were affected positively and negatively, respectively, by the abundance of Aquificae. The regression coefficients of the prediction model of Crenarchaeota were negative for PC1, and positive for PC3 and PC4 (Supplementary Table 3). These principal components were interpreted by examining the factor loadings of the PCA for environmental parameters (Supplementary Figure 3). The PC1 score increased as pH increased, and the PC1 score decreased as the concentrations of many cation species increased (Supplementary Figure 3A). The PC3 score increased as the TON or TOC increased (Supplementary Figure 3C), and the PC4 score increased as the sulfuric acid concentration increased (Supplementary Figure 3D). These results indicated that Aquificae dominated in a low-nutrient environment with high pH, low cation concentration and low TON and TOC; while Crenarchaeota dominated in a nutritious environment with high sulfuric acid concentration and with high TON and TOC. Thus, the quantitative prediction by our models supported the association between the environmental factors and community structures visualized in Figure 3.

### Enrichment Analysis of Functional Genes

**Figure 4:**
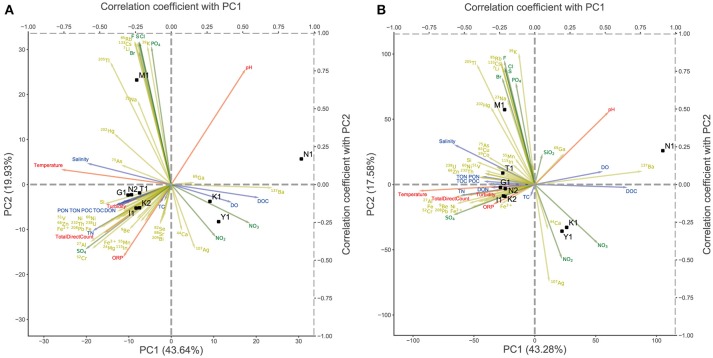
Principal component analysis (PCA) of the abundance of gene categories for each sample across nine sites. The arrows represent the correlation coefficient between the principal component scores and each environmental parameter. The yellow arrows represent cations, the green arrows represent anions, the blue arrows represent water quality and the red arrows represent physical properties. **(A)** PCA of the KEGG module abundance. **(B)** PCA of the KEGG Orthology abundance.

To investigate the comprehensive ecological features of the microbiome in the Kirishima area, we performed a KEGG-based functional annotation of predicted CDSs in the metagenome scaffolds. We assigned 70% of all CDSs on the scaffolds to either functional categories of KEGG (Supplementary Table 3). We examined differences in gene function among samples by PCA based on the abundance of the KEGG module and KO (Figure 4). The overall distribution of samples in PCA (Figure 4) was very similar to the results based on the taxonomic composition shown in Figure 3. The nutrient-rich water samples from hot springs with high ion concentrations and high TN were well-separated from the other three samples (K1, Y1, and N1). Because the major differences among samples in Figure 4 were probably attributable to the differences in functions between Aquificae and Crenarchaeota, we examined functional genes contributing to the differences in metabolic function among the samples. To characterize the metabolic functions of these two groups, we compared the KO abundance in the water from Kirishima hot spring metagenomes to the average KO abundance of representative prokaryotic genomes Supplementary Tables 2, 6; see Analysis of Gene Enrichment in the Metagenome in Materials and Methods), and identified highly enriched KOs in the Kirishima samples (Supplementary Table 6). Supplementary Table 7 shows key genes of major metabolic pathways and their enrichment scores in the Kirishima samples. Many of these enriched genes are related to autotrophic carbon fixation; that is, the 3-hydroxypropionate/4-hydroxybutyrate (3HP/4HB) cycle, the dicarboxylate/4-hydroxybutyrate (DC/4HB) cycle, the reverse tricarboxylic acid (rTCA) cycle and the reductive acetyl-CoA pathway. Genes related to sulfate and nitrogen metabolisms were also enriched, as described later. The genes assigned to K01535_H^+^-transporting ATPase were enriched in samples K2, I1, T1, G1, and N2; this is probably an important enzyme in acidic environments to maintain intracellular pH (Vanderheyden et al., 1996). The genes encoding enzymes for regulating heavy metal concentrations in cells, including K07787_cusA, silA_Cu(I)/Ag(I) efflux system membrane protein and K02006_cbiO_cobalt/nickel transport system ATP-binding protein, were enriched only in K1 and N1. The gene encoding the heat-stable and bifunctional enzyme fructose 1,6-bisphosphate aldolase/phosphatase for gluconeogenesis (K01622) (Takami et al., 2012) was abundant in all samples, except in sample Y1 (Supplementary Tables 6,7). The gene (K01906_bioW) involved in the biosynthesis of biotin required the gluconeogenesis and autotrophic pathways was also enriched in K1, Y1 and N1 (Supplementary Table 7).

Various autotrophic carbon-fixing pathways, which produce organic matter from CO_2_, have been reported in extreme environments (Hügler and Sievert, 2011). Genes (K15230_aclA, K15231_aclB) encoding ATP-citrate lyase, a key enzyme involved in determining the direction and citric acid cleavage of the rTCA cycle (Aoshima et al., 2004), were enriched in three samples (K1, Y1, and N1) but were not detected in other samples (Supplementary Table 7). The genes encoding enzymes for an alternative citrate cleavage pathway in the rTCA cycle (K15232_ccsA, K15233_ccsB, and K15234_ccl) were also found abundantly in K1, N1, and M1 but not detected in Y1 (Supplementary Table 7). These results suggest that the rTCA cycle is the primary pathway in the samples with relatively high transparency, low ion concentrations and low nutrition. The genes involved in this pathway are also enriched in a deep-sea chimney microbiome and considered to be an important CO_2_ fixation pathway in the environment (Nakagawa and Takai, 2008; Xie et al., 2011).

The genes (K14465_succinate semialdehyde reductase, K14534_abfD) required for both the DC/4-HB and 3-HP/4-HB cycles (Scherf and Buckel, 1993) and the genes encoding K14467_4-hydroxybutyryl-CoA synthetase and K14466_4-hydroxybutyryl-CoA synthetase, which are characteristic of the DC/4-HB (Hawkins et al., 2013) and 3-HP/4-HB cycles, respectively, were enriched in samples that were highly turbid and had high nutrition (M1, K2, I1, T1, G1, and N2). Our data support a previous report based on transcriptomic analysis of microbiota in terrestrial hot springs of Tengchong, China (Li et al., 2010), which suggested that both cycles play important roles in nutrition-rich environments (Supplementary Table 7). Because genes (K14468_mcr, K14469 acrylyl-CoA reductase) responsible for the 3-Hydroxypropionate (3HP) bi-cycle (Zarzycki et al., 2009) were not found, the 3HP bi-cycle may not be necessary in the Kirishima hot spring water ecosystem.

Key genes coding the acetyl-CoA decarboxylase/synthase complex are required for the reductive acetyl-CoA pathway, each of which is classified into “bacteria type” and “archaea type” (Takami et al., 2012). The bacteria-type genes (e.g., K00198_cooS, acsA, K14138_acsB) for the ACDS complex were highly abundant in samples N1 and K1, while the archaea-type genes (e.g., K00192_cdhA and K00194_cdhD) were low in abundance. As reported in studies of deep-sea hydrothermal vents (Xie et al., 2011; Takami et al., 2012), the anaerobic ACDS complex might be used by anaerobic microorganisms to couple CO oxidation to sulfate reduction, or to reduce CO_2_ to acetate in the Kirishima hot spring water ecosystem. With respect to other carbon-fixation pathways, the gene encoding ribulose bisphosphate carboxylase/oxygenase (RuBisCO) (K01601_rbcL and K01602_rbcS), the key enzyme of the Calvin cycle, was found only rarely in the samples. This suggests that this cycle is not a major metabolic pathway in the Kirishima hot spring water ecosystem.

Because water from hot springs generally contains sulfur compounds, sulfate metabolism is important for energy conservation in these extreme environments (Xie et al., 2011). The genes (K17222_soxA1, K17223_soxX, K17224_soxB1, K17226_soxY, and K17227_soxZ) coding the sulfur-oxidizing multienzyme system (sox), related to oxidation of thiosulfate (Friedrich et al., 2005), were frequently detected in samples with low nutrition (e.g., K1 and N1) (Supplementary Table 7), in agreement with previous metagenomic analyses of YNP hot springs and deep-sea hydrothermal vents (Inskeep et al., 2010; Xie et al., 2011). Also, with respect to other sulfuric acid pathways, K16952_sor, which is involved in thiosulfate oxidation via the elemental sulfur metabolism (Friedrich et al., 2005), was abundant in the Y1, K2, I1, T1, G1, and N2 samples, but not in K1 and M1. One of the genes (K17218_sqr) encoding sulfide-quinone oxidoreductase (SQR) involved in sulfur oxidation by reducing sulfide (Gregersen et al., 2011) was detected at a high abundance in all samples (Supplementary Table 7). These data suggested the existence of these sulfur-reducing and oxidizing abilities via SOX and SQR systems in the Kirishima hot spring water ecosystem.

Dissimilatory sulfate reduction and oxidation can be involved in both energy conservation and sulfite metabolism via the adenylyl sulfate (APS) pathway with ATP production (Friedrich et al., 2005). In the Kirishima metagenomes, a set of genes (K11180_dsrA, K11181_dsrB, K00394_aprA, K00395_aprB) involved in this pathway was highly abundant in all samples, except in Y1 (Supplementary Table 7). High concentrations of sulfate ions were observed in several samples (K2, I1, T1, G1, and N2). A set of genes (K16933_doxB, K16936_doxA, K16934_doxC, and K16935_doxE) involved in oxidation of thiosulfate via tetrathionic acid was found in most Crenarchaeota (Müller et al., 2004) and was highly represented in K2, I1, T1, G1, and N2 (but not in Y1), depending on the high sulfate ion properties of the sample sites. As seen in *Sulfolobus* in a previous study (Quehenberger et al., 2017), some facultative autotrophs (e.g., *Sulfolobus* dominating sample N2) that can oxidize reduced sulfur compounds via various sulfur oxidation pathways may exist in the Kirishima environments.

Hydrogen metabolism is considered to be one of the most important processes in hydrogen-containing alkaline environments, such as serpentinite-hosted ecosystems (Brazelton et al., 2012). In the Kirishima metagenomes, however, the abundances of genes responsible for hydrogen metabolism (K05587_hoxF_bidirectional [NiFe] hydrogenase, K05588_hoxU_bidirectional [NiFe] hydrogenase, K18008_hydA2_[NiFe] hydrogenase) was low or not detected. Hydrogen metabolism cannot be crucial for the Kirishima ecosystem because hydrogen was below the detection limit in all samples (data not shown).

The importance of denitrification, nitrogen fixation and nitrification constituting the nitrogen cycle has been suggested by studies of deep-sea hydrothermal environments (Tamegai et al., 2007). In the Kirishima samples with high transparency and low nutrition, genes involved in denitrification were much more abundant than those for nitrogen fixation and nitrification (Supplementary Table 7). The gene (K02567_napA) associated with nitrate reduction (denitrification and dissimilatory nitrate reduction) was frequently found in samples N1 and K1 (Supplementary Table 7). Likewise, gene (K00367_narB) for assimilatory nitrate (nitrite) reduction (Morozkina and Zvyagilskaya, 2007) was found in high proportions in M1, N1, and K1. In general, the oxidation of sulfur compounds can be coupled to the reduction of electron acceptors including oxygen and nitrate (Brunet and Garcia-Gil, 1996). These data imply that nitrate reduction, such as denitrification coupled to sulfur oxidation is likely to be an important energy-generating pathway fueling the microbial communities in the Kirishima hot spring water ecosystem.

However, the gene encoding nitrogenase reductase (K02588_nifH) for nitrogen fixation and the gene related to nitrate oxidation (K00368_nirK) were rarely found in any samples. These results indicate that nitrification is not a major metabolic pathway in the Kirishima hot spring water ecosystem. The reliability of the gene enrichment analysis was also supported by the result of the correlation analysis between coordinates of samples on the PCA plot and measured values of environmental factors (Figure 4), in which the arrow of the nitrate ions directed to the position of sample K1.

In this study, based on the metagenomic and geochemical data, we clarified that the microbial ecosystem of the Kirishima hot spring water was dominated by either Crenarchaeota or Aquificae. These two types of microbial community structures, and the gradient of relative abundance between them, were strongly affected by their habitat-specific environmental factors, as suggested by both the PCA and regression analyses. The key environmental factors (such as sulfate and nitrate ions) identified by these analyses were consistent in the enrichment analysis of gene functions, especially for the sulfur and nitrogen metabolisms and autotrophic carbon fixation. These factors are probably crucial for the formation of specific community structural patterns. Compared with ocean or soil environments, many environmental factors impose severe restrictions on survival in extreme environments, so it is likely that specific community structures tend to be formed as a result of adaptation, according to the metabolic features of each microorganism.

Because the hot springs in the Kirishima area show diverse chemical properties and represent a wide variety of hot spring environments, our finding in the Kirishima ecosystem (i.e., the relationship between microorganisms and environmental parameters) may be applied to many other hot spring environments. To test the generality of our findings, we also investigated microbial and environmental data in the same way for a hot spring in Hachijo-jima, an island 850 km from Kyushu. Water from the Hachijo-jima hot spring is both low in nutrition and transparent (Supplementary Table 4) and, compared with the Kirishima samples, its microbial community was seen to belong to a group dominated by Aquificae (Supplementary Figure 4). This result suggests that the relationship we found between the environmental parameters and the microbial community structures could be a general pattern applicable to hot spring environments other than those in the Kirishima area. Our study shows that investigation of the chemical properties of hot springs makes it possible to predict, to some extent, the taxonomic composition and metabolic characteristics of the microorganisms living in the extreme environment. These data will provide clues to assist the more efficient exploration of biological resources, such as useful enzymes and bioactive compounds and lead to potential biotechnology applications.

Enrichment analysis among samples in the Kirishima area revealed that genes encoding autotrophic carbon fixation, such as the 3HP/4HB cycle, the DC/4HB cycle and the reductive acetyl-CoA pathway, were detected at a high abundance in the nutrition-rich samples. On the other hand, genes encoding the rTCA cycle were detected in high proportions only in the samples with low nutrition. The reason for the variations in these functions is presumed to be the difference between the dominant taxa; Crenarchaeota may use the 3HP/4HB or DC/4HB cycle, and Aquificae may use the rTCA cycle, considering information on cultured strains (Hügler and Sievert, 2011). In addition, genes related to various sulfate metabolic pathways were enriched in all samples. Previous metagenomic studies of the YNP hot springs and hydrothermal vent chimney 4143-1 also revealed a high abundance of genes involved in sulfur metabolism (Inskeep et al., 2010; Xie et al., 2011). Sulfate utilization is the main mechanism of energy acquisition, and may be crucial for the survival of sulfate-oxidizing and reducing facultative autotrophs in Kirishima and other hot springs. The presence of the key genes of carbon fixation and sulfur oxidation pathways detected in the metagenomes of water from the Kirishima hot springs implies that both obligate and facultative autotrophs are present, and contribute to biomass production in the Kirishima hot spring water ecosystem.

The unique environmental features of the Kirishima area provide us an opportunity to investigate diverse hot spring ecosystems in proximal locations. Further studies of microbial ecosystems in various extreme environments (including the Kirishima area), using detailed environmental data, will provide valuable information that will assist in the prediction of microbial ecosystems from environmental data, and the exploration of biological resources in extreme environments.

## Data Availability

Metagenomic sequence reads have been deposited in the DNA Databank of Japan (DDBJ) with BioProject ID PRJDB7293.

## Author Contributions

EN, KS, SO, SM, YH, and KK designed the research. EN, KS, HN, SM, YH, and KK collected the samples. EN, KS, HN, and SO performed biological and chemical experiments. EN, KH, and HM performed *in silico* analysis. EN, KH, HM, YH, and KK wrote the manuscript.

### Conflict of Interest Statement

EN was employed by FASMAC Co., Ltd. The remaining authors declare that the research was conducted in the absence of any commercial or financial relationships that could be construed as a potential conflict of interest.
