# Supplementary material for: The Relationship Between Microbial Community Structures and Environmental Parameters Revealed by Metagenomic Analysis of Hot Spring Water in the Kirishima Area, Japan
Source: Front Bioeng Biotechnol. 2018 Dec 20;6:202. doi: 10.3389/fbioe.2018.00202 (PMC6306410; doi:10.3389/fbioe.2018.00202)
Supplement: Supplementary file 1 [file Data_Sheet_1.pdf]

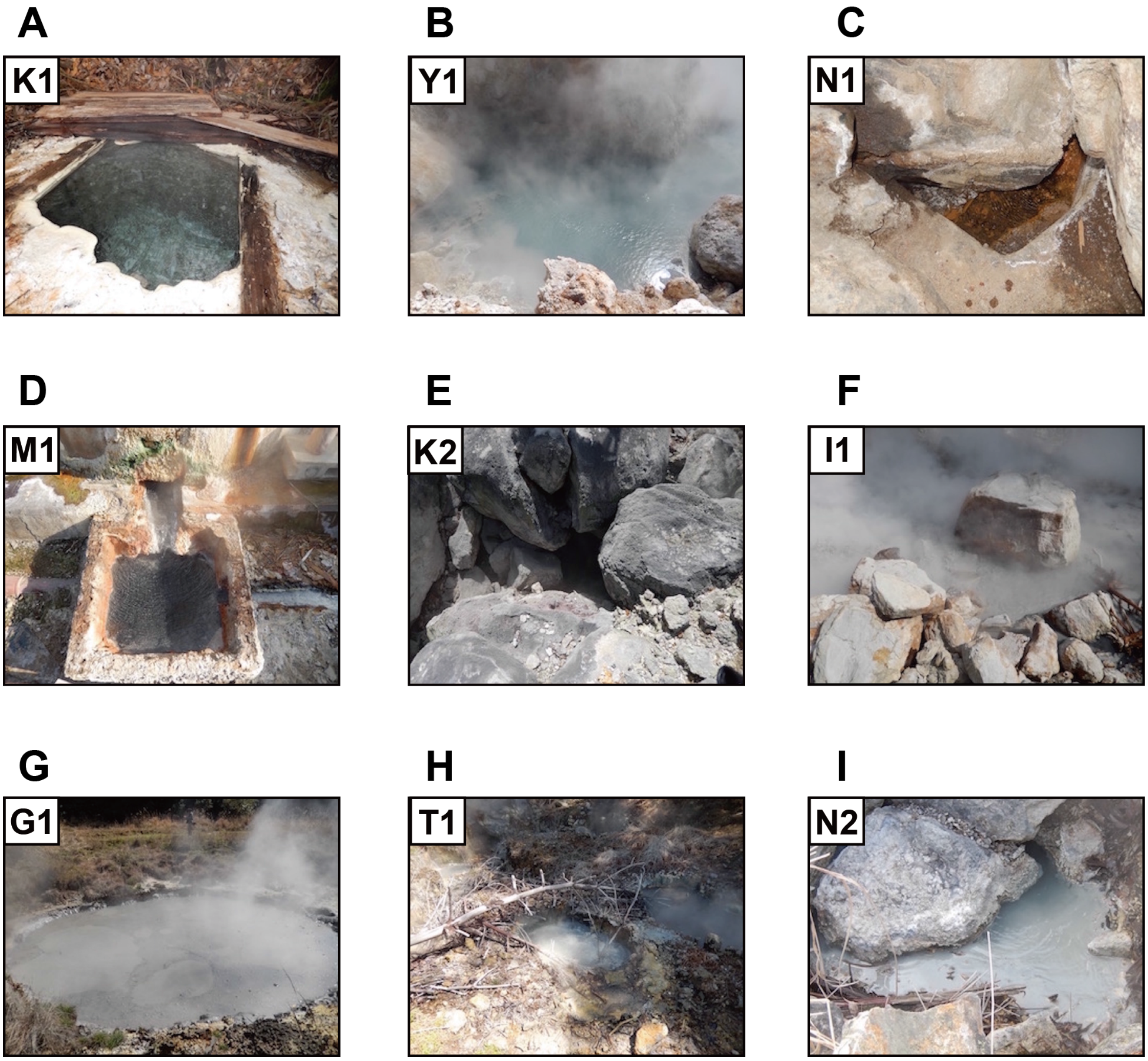

Supplementary Figure 1. Sampling sites in the Kirishima area, Japan. Site names are spelled out in Figure 1.
