# Supplementary material for: The Relationship Between Microbial Community Structures and Environmental Parameters Revealed by Metagenomic Analysis of Hot Spring Water in the Kirishima Area, Japan
Source: Front Bioeng Biotechnol. 2018 Dec 20;6:202. doi: 10.3389/fbioe.2018.00202 (PMC6306410; doi:10.3389/fbioe.2018.00202)
Supplement: Supplementary file 2 [file Data_Sheet_2.pdf]

A

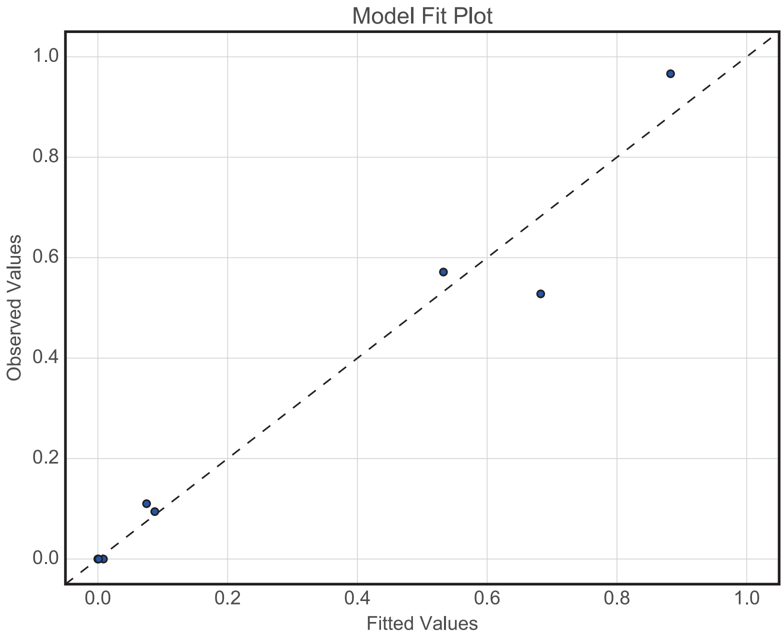

B

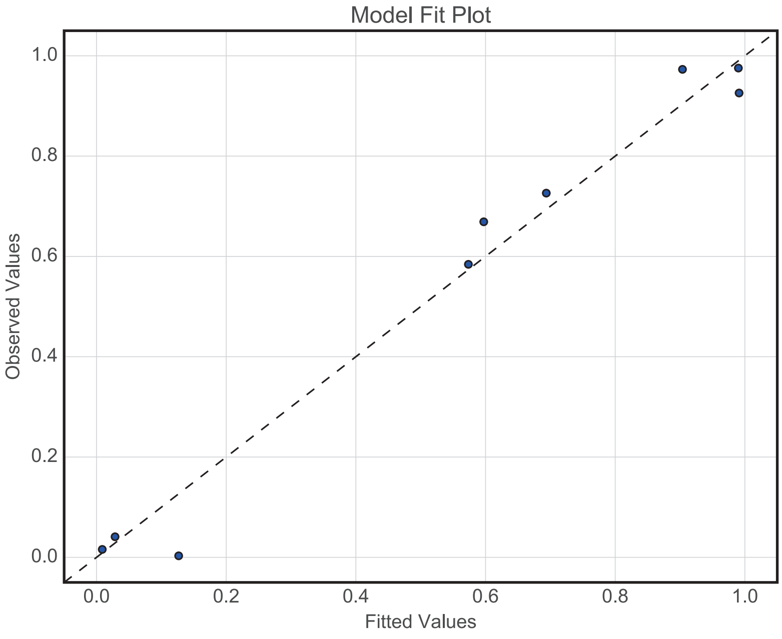

Supplementary Figure 2. Comparison between observed relative abundances and fitted values by the regression models among nine samples. A. Results of the model to predict relative abundance of Aquificae. B. Results of the model to predict relative abundance of Crenarchaeota.
