# Supplementary material for: The Relationship Between Microbial Community Structures and Environmental Parameters Revealed by Metagenomic Analysis of Hot Spring Water in the Kirishima Area, Japan
Source: Front Bioeng Biotechnol. 2018 Dec 20;6:202. doi: 10.3389/fbioe.2018.00202 (PMC6306410; doi:10.3389/fbioe.2018.00202)
Supplement: Supplementary file 3 [file Data_Sheet_3.pdf]

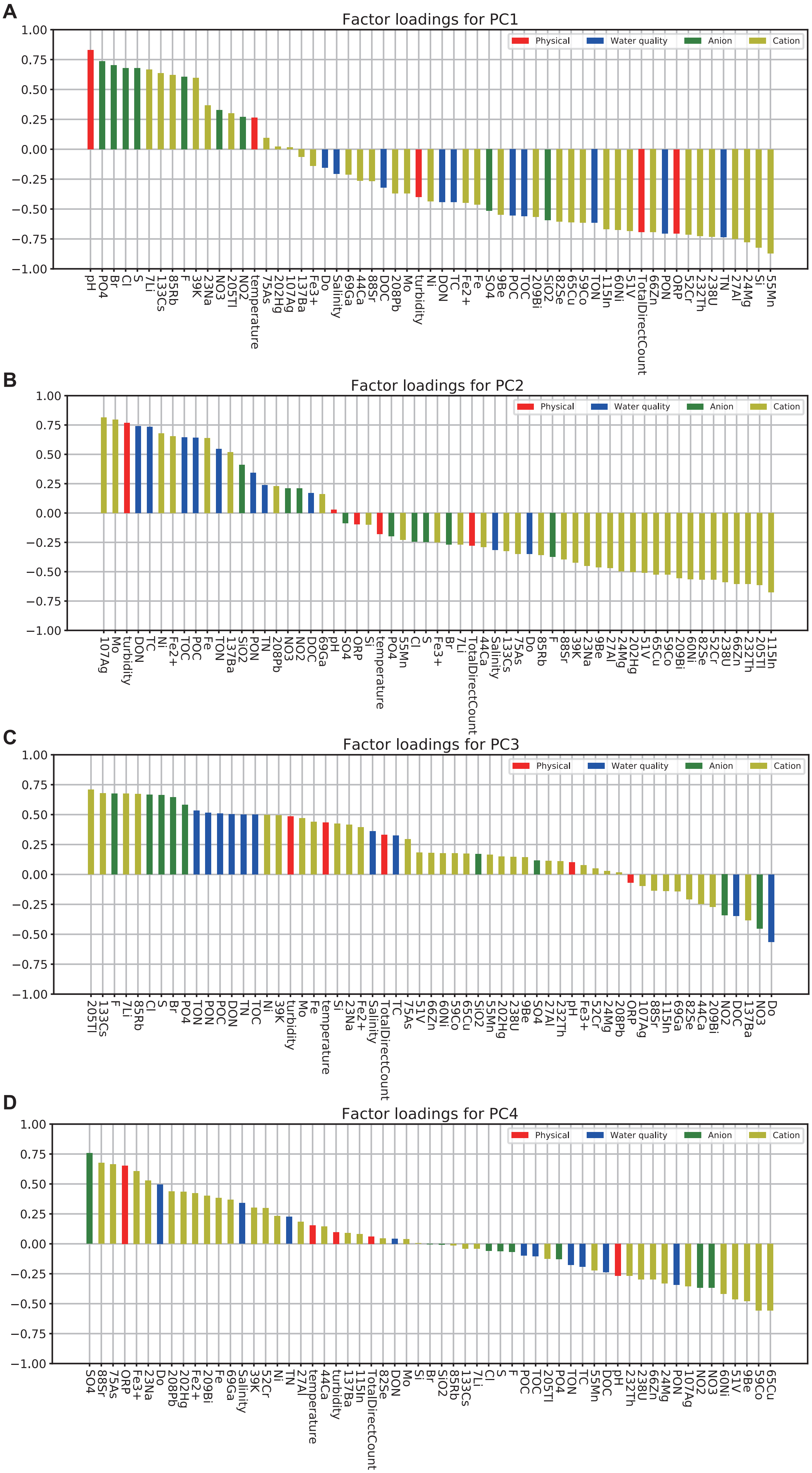

Supplementary Figure 3. Factor loarding by principal component analysis of environmental data. A. Factor loarding for PC1. B. Factor loarding for PC2. C. Factor loarding for PC3. D. Factor loarding for PC4.
