# Supplementary material for: The Relationship Between Microbial Community Structures and Environmental Parameters Revealed by Metagenomic Analysis of Hot Spring Water in the Kirishima Area, Japan
Source: Front Bioeng Biotechnol. 2018 Dec 20;6:202. doi: 10.3389/fbioe.2018.00202 (PMC6306410; doi:10.3389/fbioe.2018.00202)
Supplement: Supplementary file 4 [file Data_Sheet_4.pdf]

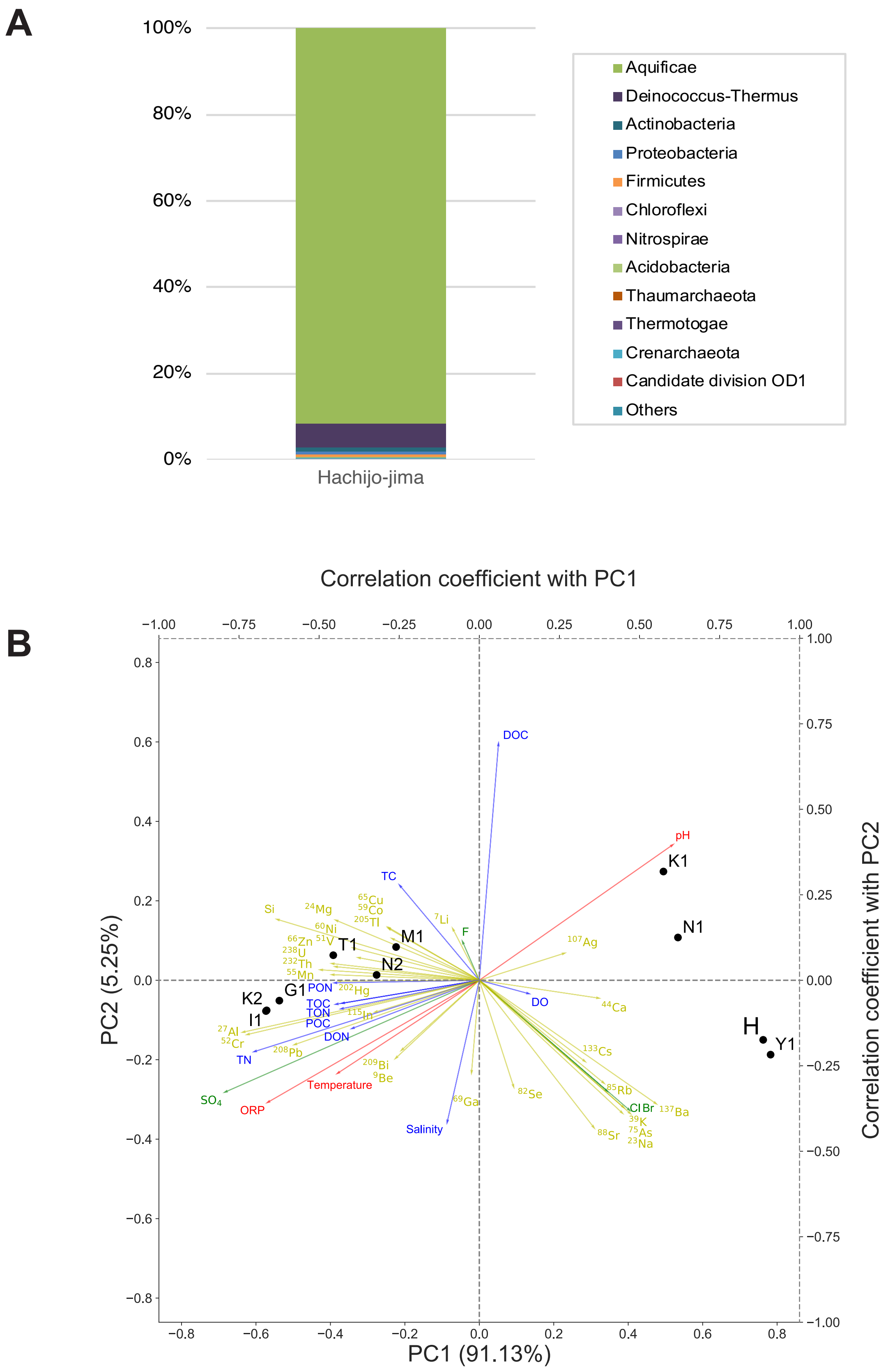

Supplementary Figure 4. Taxonomic composition of microbiota in hot spring from Hachijo-jima, Japan, and principal component analysis (PCA) of the relative abundance of each sample across ten sites, including Hachijo-jima. A. Taxonomic composition of the microbiota at phylum level at Hachijo-jima sampling point (33°04′27.9″ N, 139°48′43.4″ E) based on 16S rRNA genes identified from the metagenomic reads. B. PCA of the relative abundance of each sample across ten sites, including Hachijo-jima (abbreviated as H). Yellow arrows, cations; green arrows, anions; blue arrows, water quality; red arrows, physical properties.
