# Supplementary material for: The Relationship Between Microbial Community Structures and Environmental Parameters Revealed by Metagenomic Analysis of Hot Spring Water in the Kirishima Area, Japan
Source: Front Bioeng Biotechnol. 2018 Dec 20;6:202. doi: 10.3389/fbioe.2018.00202 (PMC6306410; doi:10.3389/fbioe.2018.00202)
Supplement: Supplementary file 5 [file Table_1.docx]

Supplementary Material

The relationship between microbial community structures and environmental parameters revealed by metagenomic analysis of hot spring water in the Kirishima area, Japan

**Eri Nishiyama*, Koichi Higashi, Hiroshi Mori, Konomi Suda, Hitomi Nakamura, Soichi Omori, Shigenori Maruyama, Yuichi Hongoh, Ken Kurokawa**

*** Correspondence:** Eri Nishiyama: enishiyama@fasmac.co.jp

# Supplementary Figures and Tables

## Supplementary Figures

**Supplementary Figure 1.** Sampling sites in the Kirishima area, Japan. Site names are spelled out in Figure 1.

**Supplementary Figure 2.** Comparison between observed relative abundances and fitted values by the regression models among nine samples. A. Results of the model to predict relative abundance of Aquificae. B. Results of the model to predict relative abundance of Crenarchaeota.

**Supplementary Figure S3.** Factor loading by principal component analysis of environmental data. A. Factor loading for PC1. B. Factor loading for PC2. C. Factor loading for PC3. D. Factor loading for PC4.

**Supplementary Figure 4.** Taxonomic composition of microbiota in hot spring from Hachijo-jima, Japan, and principal component analysis (PCA) of the relative abundance of each sample across ten sites, including Hachijo-jima. A. Taxonomic composition of the microbiota at phylum level at Hachijo-jima sampling point (33°04′27.9″ N, 139°48′43.4″ E) based on 16S rRNA genes identified from the metagenomic reads. B. PCA of the relative abundance of each sample across ten sites, including Hachijo-jima (abbreviated as H). Yellow arrows, cations; green arrows, anions; blue arrows, water quality; red arrows, physical properties.

## Supplementary Tables

**Supplementary Table 1.** Samples from hot spring samples investigated in this study

**Supplementary Table 2.** Strains selected for the enrichment analysis

**Supplementary Table 3.** Regression analysis to predict abundance of Aquificae and Crenarchaeota

**Supplementary Table 4.** Physical, chemical and biological properties of samples from hot springs

**Supplementary Table 5.** Statistics of metagenomic sequencing data

**Supplementary Table 6.** Enrichment scores for gene categories (classification based on KEGG Orthology: KO) identified in the Kirishima hot spring metagenomes

**Supplementary Table 7.** Enriched gene categories and genes coding key metabolic enzymes from the Kirishima hot spring metagenomes, collated from Supplementary Table 6.
